# Comic-Based Educational Intervention Enhances Antimicrobial Resistance Knowledge and Perceptions Among Adolescents in Ghana

**DOI:** 10.3390/antibiotics15070646

**Published:** 2026-06-29

**Authors:** Obed Kwabena Offe Amponsah, Marie Millicent Baffoe-Bonnie, Annabella Bensusan Osafo, Nana Akua Abruquah, Emmanuel Konadu, Douglas Aninng Opoku, Charlotte Boachie Danquah, Benedicta Bosu, Evans Owusu-Ansah, Nana Kwaku Bugyei Buabeng, Aaron Courtenay, Ahmed Abuelhana, Kwame Ohene Buabeng, Nana Kwame Ayisi-Boateng

**Affiliations:** 1 Department of Pharmacy Practice, Kwame Nkrumah University of Science and Technology, Kumasi 00233, Ghana; 2 Komfo Anokye Teaching Hospital, Kumasi 00233, Ghana; 3 University Hospital, Kwame Nkrumah University of Science and Technology, Kumasi 00233, Ghana; 4 University of North Texas Health at Fort-Worth, 3500 Camp Bowie Blvd, Forth Worth, TX 76107, USA; 5 Centre for International Health Innovation and Partnership (CIHIP), University of Ulster, Northern Ireland, Coleraine BT52 1SA, UK; 6 School of Pharmacy, University of Health and Allied Sciences, Ho 00233, Ghana

**Keywords:** antimicrobial resistance, AMR education, adolescents, comics, Ghana, school-based intervention visual storytelling, antimicrobial stewardship

## Abstract

**Background**: Antimicrobial resistance (AMR) is a major global health threat, yet awareness among adolescents remains low, especially in low-resource settings. Novel educational approaches are needed to appropriately inform and sustain the interest of young people to encourage behavioral change in the use of antimicrobials. This study aimed to evaluate the impact of a comic-based intervention in improving AMR knowledge and perceptions regarding the responsible use of antimicrobials among junior high school students in Ghana. **Methods**: We conducted a before-and-after educational intervention at the Kwame Nkrumah University of Science and Technology (KNUST) Basic Schools in Kumasi, Ghana, during the 2023 World AMR Awareness Week. All junior high students (JHS 1–3) present were exposed to a comic storyline illustrating appropriate antibiotic use and the risks of AMR, delivered via PowerPoint presentation and print, as an intervention. Questionnaires were administered pre- and post-intervention to assess changes in AMR knowledge and perceptions. **Results**: The median age of the student participants was 13 years. Out of the 1068 students involved, 611 matched responses were analyzed. Knowledge of AMR increased significantly after the comic intervention (*p* < 0.001). Correct responses increased across all items, and the proportion of students with a knowledge score increased substantially (33% to 70%). Attitudinal perceptions also shifted positively: 85.1% acknowledged their personal role in tackling AMR after the intervention, up from 67.4% before the intervention (*p* < 0.001). **Conclusions**: The educational intervention resulted in increased AMR knowledge and positive perceptions among the adolescents. The study findings suggest that comics and their deployment are viable tools for enhancing AMR awareness in school settings. Therefore, integrating and engaging visual storytelling in health education programs can increase awareness and promote responsible behavior towards the use of antimicrobials and support the global and national efforts for the containment of AMR.

## 1. Introduction

The effectiveness of medical treatments, such as surgeries, cancer therapies, and treatments for infectious diseases, could be negatively affected by the rise in antimicrobial resistance (AMR), which poses substantial risks to public health [1]. It is a global epidemic that requires immediate attention from both healthcare professionals and the public, particularly in low- and middle-income nations, where the burden of infectious diseases remains significantly high [2,3].

Despite its importance, public awareness and understanding of AMR remain low, particularly among young people and those with limited education [4,5]. This information gap is especially significant for adolescents [6], who are at a key stage for developing health-related behaviors and attitudes. Some studies have shown, for instance, that behavioral and biological risk factors for non-communicable diseases are formed in childhood and adolescence and remain until adulthood [7,8,9,10]. There is a dearth of focused educational initiatives to inform adolescents about the risks of AMR and the significance of safe antimicrobial use in countries like Ghana. Bridging this gap is essential to developing a generation of knowledgeable people who will advocate for and practice good antimicrobial use.

Conventional approaches to teaching AMR, such as lectures, workshops or written materials, have frequently failed to keep students’ attention, particularly in environments with limited educational resources and short attention spans [11]. In recent years, there has been an increasing interest in using novel techniques for teaching health, one of which is the use of comics [12,13,14]. Comics are increasingly recognized as a useful medium for communicating complex concepts in an interesting and easily digestible fashion, particularly for younger audiences. Studies have demonstrated that visual storytelling, along with written explanations, can dramatically improve students’ knowledge and recall of health-related content [12,13,14].

Such innovative means of educating and creating awareness could be very useful for AMR education, especially among young people. This means that health education could increase the capacity of beneficiaries to act in ways that would reduce the development and progression of AMR. This knowledge could also lead to behavior changes critical for AMS [15]. In Ghana, as in many other regions throughout the world, the use of comics in health education remains underexplored. This study seeks to fill this gap by examining how comics can be used to enhance the knowledge and attitudes regarding AMR among adolescents in junior high school, providing valuable insights into how visual media can be utilized in health education in resource-constrained settings. As part of the antimicrobial stewardship (AMS) activities at the University Hospital of Kwame Nkrumah University of Science and Technology (KNUST), an awareness-creation exercise was conducted at the Basic School of KNUST in November 2023. This study aims to contribute to the broader effort of combating AMR through innovative educational strategies that resonate with younger audiences.

## 2. Results

**Figure 1:**
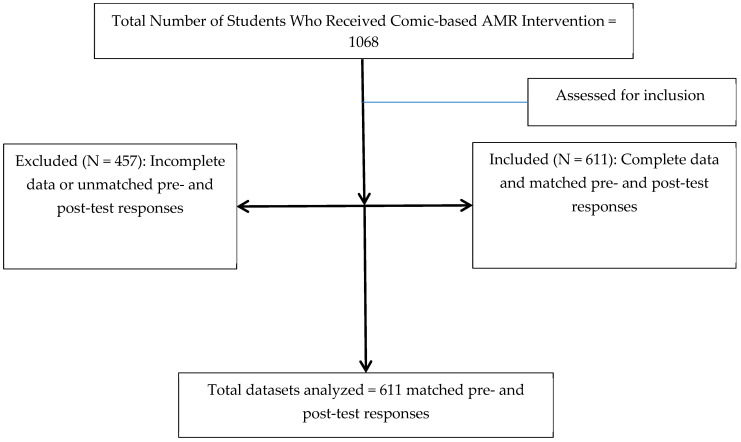
Flowchart showing distribution of included and excluded responses of participants that took part in the educational intervention at the University Basic School, KNUST.

A total of 1068 students were given educational intervention and administered pre- and post-test questionnaires. The number of responses included in the analyses was 611, as shown in Figure 1.

### 2.1. Sociodemographic Characteristics of Respondents

The median age of the respondents was 13 years, with more than one-third (37.32%) of the respondents in JHS 1. More than half (55.65%) of the respondents were females. Regarding their living situation, almost ninety-four percent of the respondents resided with their parents. The parents’ median age was 46 and 41 years for the father and mother, respectively. More than three-quarters (75.89%) of the respondents’ fathers had a tertiary education, and nearly two-thirds (64.45%) of the respondents’ mothers also obtained a tertiary education as the highest educational qualification. One-quarter of respondents residing with their guardians had guardians with a tertiary education (Table 1).

### 2.2. Antibiotic Resistance Awareness and Source of Information

More than ninety percent (94.58%) of the respondents were aware of antibiotic resistance. Health workers (61.0%) were the most cited source of information concerning AMR, followed by an educational campaign (30%). Ninety-three percent (93.0%) of the respondents knew the definition of antibiotic resistance. Two-thirds (65.58%) of the respondents had first-degree family members who were healthcare professionals (Table 2).

### 2.3. Perception of Respondents Towards Antimicrobial Use

Concern about antibiotic resistance showed a significant increase in borderline cases, with positive responses rising from 71.0% for the pre-test to 75.6% for the post-test (*p* = 0.050). Furthermore, awareness of antibiotic resistance as a global issue remained essentially unchanged, with 73.0% of participants in the pre-test and 75.5% in the post-test recognizing its global nature (*p* = 0.3). Similarly, perceptions of the limited impact of antibiotic resistance showed no significant variation, with positive responses remaining stable at 64.2% pre-intervention and 63.3% post-intervention (*p* = 0.8). A considerable increase in perception scores was observed regarding participants’ responsibility in mitigating antibiotic resistance. Positive responses increased from 67.4% for the pre-test to 85.1% for the post-test (*p* < 0.001). These are shown in Table 3.

### 2.4. Knowledge of Respondents on Antimicrobial Resistance

After the educational intervention, knowledge was significantly changed across all questions assessing antimicrobial resistance. Regarding antibiotic resistance, there was a significant increase in knowledge in that domain from 59.2% to 78.4%, *p*-value < 0.001. Also, there was more than a quarter increase in the understanding of the risks of self-medicating with antibiotics without consulting a health professional or purchasing them without a prescription (57.6% to 83.6%, *p*-value < 0.001). There was also a substantial increase in knowledge about the risks of using leftover antibiotics from previous infections, rising from 47.6% to 76.9% (*p* < 0.001). Knowledge regarding the risk of treatment failure due to not completing a course of antibiotic therapy increased by nearly 20 percentage points from 70.9% to 90.5% (*p* < 0.001), as shown in Table 4.

### 2.5. Knowledge of Respondents on Antibiotic Use

The intervention led to a significant increase in respondents’ knowledge scores on antibiotic use. Familiarity with antibiotics showed a marked increase, with correct responses rising from 43.7% for the pre-test to 94.8% for the post-test (*p* < 0.001). Awareness that antibiotics are ineffective against viral infections also increased significantly, with correct responses increasing from 34.4% to 56.1% (*p* < 0.001). Similarly, the proportion of respondents correctly identifying that antibiotics are not appropriate for treating coughing and wheezing rose from 39.8% for the pre-test to 59.6% for the post-test (*p* < 0.001). Knowledge about the spread of antibiotic-resistant bacteria also demonstrated significant gains, with correct responses increasing from 46.3% to 69.4% (*p* < 0.001). The understanding that low-dose antibiotics are not beneficial increased significantly, with correct responses rising from 38.3% for the pre-test to 44.8% for the post-test (*p* = 0.007), as shown in Table 5.

### 2.6. Overall Knowledge of Participants Before and After Intervention

The educational intervention led to a significant increase in knowledge scores across the knowledge levels. The McNemar test showed significant overall shifts in knowledge scores with a moderate effect size of 0.38. Among participants who initially had scores categorized as poor, 41% significantly increased to moderate, and 24% significantly increased to good (*p* ≤ 0.001). For those in the moderate category, 38% significantly increased to good (*p* ≤ 0.001). Among participants in the good category, the majority (62%) retained their good scores, with a small but significant decline of 34% to moderate (*p* ≤ 0.001), as shown in Table 6.

### 2.7. Determinants of Knowledge on Antimicrobial Use and Antimicrobial Resistance

In the simple linear regression model shown in Table 7, being in JHS 2 was associated with a significant increase in knowledge relative to being in JHS 1 (β = 0.66, 95% CI: 0.24, 1.1, and *p* = 0.002). Male respondents had significantly lower knowledge scores than females (β = −0.67, 95% CI: −1.0, −0.32, and *p* < 0.001). Media sources such as TV, radio, and social media were associated with lower knowledge scores compared to those who did not report these sources (β = −0.57, 95% CI: −1.1, −0.05, and *p* = 0.033).

### 2.8. Multivariable Linear Regression Analysis

In the multivariable linear regression model, gender and information sources remained significant. Male respondents continued to demonstrate lower knowledge scores than females (β = −0.51, 95% CI: −1.0, −0.01, and *p* = 0.045). Media as a source of information was still associated with lower knowledge scores (β = −0.56, 95% CI: −1.1, −0.04, and *p* = 0.035). Educational campaigns were also found to negatively influence knowledge scores (β = −1.4, 95% CI: −2.6, −0.17, and *p* = 0.025), as shown in Table 8.

## 3. Discussion

We used visual storytelling with comics as an educational intervention to increase knowledge and perception scores in relation to AMR and antibiotic use among junior high school students at a University Basic School in the Ashanti region of Ghana. To the best of our knowledge, this study contributes to the limited body of literature showing an intervention targeting adolescents using comics with a demonstrated increase in knowledge and perceptions towards AMR and antibiotic use in the African region.

Almost all the respondents reported being aware of AMR in addition to knowing the definition of AMR. Health workers were cited as a source of information concerning AMR by almost two-thirds of the students, followed by a third citing educational campaigns. Two-thirds of the respondents had first-degree family members who were healthcare professionals, which may account for the high level of awareness of AMR among the respondents. Further, the students included in this study are within a few months to three years of finishing JHS and are expected to have a potentially good level of inferential thinking, which may facilitate the deduction of the term. In a different study assessing trusted sources of health information online, health workers (personal doctors and medical universities) were the most trusted sources of information among participants. Similar to this current study, individuals trusting their doctor were found to be younger in that study [16]. Health workers may thus be a good source of information regarding AMR that could be leveraged to increase the knowledge of the public and especially young people.

Concern about AMR showed a statistically significant increase from the baseline, with positive responses rising by 4.6% from the pre-test to the post-test. However, the recognition of AMR as a global issue remained essentially unchanged between the pre-test and post-test. Similarly, incorrect perceptions that AMR has a limited impact showed no significant variation between the pre-test and post-test. This is a target for improvement of the educational tool to better cover this perception, as it may potentially impact their adherence to action points towards mitigating AMR. The storyline of the comics may need to be updated to include information on the global and one-health nature of AMR infections, as well as the potential for spreading to others and the environment. Notwithstanding this, a considerable positive increase was observed in participants acknowledging their responsibility in mitigating antibiotic resistance. Positive responses increased by 17.7% from the pre-test to the post-test. Another study in adults piloted an online digital intervention to reduce the inappropriate demand for antibiotics and was successful in improving patient beliefs about antibiotics and AMR [17]. This highlights the utility of interactive activities like the current intervention towards improving positive perceptions about AMR, particularly in young people.

The educational intervention led to a significant increase in knowledge scores across the knowledge levels assessed as poor, moderate, or good. Participants significantly increased their overall knowledge levels from the pre-test to the post-test. This may likely be due to the interactive nature of the intervention, which has been shown in another study to improve knowledge among participants [18]. Regarding antibiotic resistance, there was a significant increase in knowledge in that domain for the post-test. All aspects assessed under this domain on resistance changed positively from the pre-test to the post-test. This educational intervention using comics among these students was able to increase knowledge scores about antimicrobial resistance in a statistically significant manner.

The intervention also led to a significant increase in respondents’ knowledge scores on antibiotic use. Familiarity with antibiotics, awareness that antibiotics are ineffective against viral infections, and knowledge about the spread of antibiotic-resistant bacteria all increased significantly in the post-test. Furthermore, the understanding that low-dose (underdosed) antibiotics are not beneficial increased significantly, with correct responses rising by 6.5% from pre-test to post-test. The understanding that underdosing antibiotics is not beneficial increased only marginally compared to the others. The storyline in the comics may not have covered this aspect of the training as much as the other elements of the knowledge domain. Future updates of the educational material could include more age-appropriate content to increase the change in knowledge than what was obtained in this study. These findings underscore substantial progress in participants’ knowledge across critical aspects of antibiotic use and resistance with room for improvement.

In the simple linear regression model, being in JHS 2 was associated with a significant increase in knowledge relative to being in JHS 1 (β = 0.66, 95% CI: 0.24, 1.1, and *p* = 0.002). This may not be surprising, as they are more advanced learners than JHS 1 and may have better deductive abilities. Male respondents had significantly lower knowledge scores than females (β = −0.67, 95% CI: −1.0, −0.32, and *p* < 0.001). Male respondents continued to demonstrate lower knowledge scores than females (β = −0.51, 95% CI: −1.0, −0.01, and *p* = 0.045) in the multivariable linear regression model. A study by Houtte (2004) also showed that males generally perform worse than females in general schools, potentially due to the culture of males being less study-oriented than females [19]. Another study has reported that boys take it easier, work less hard, and are distracted more quickly, which may account for the finding in the current study [20]. Males in future studies may thus need more encouragement to ensure they follow lessons and pay more attention to ensure learning is achieved. However, further assessments are needed to establish this or another reason as a cause for the low performance among the male students in this study.

Media sources such as TV, radio, and social media were associated with lower knowledge scores compared to those who did not report these sources in the univariate analysis (β = −0.57, 95% CI: −1.1, −0.05, and *p* = 0.033) as well as the multivariable linear regression analysis (β = −0.56, 95% CI: −1.1, −0.04, and *p* = 0.035). This may potentially support the initial assertion of increased AMR awareness as a result of health workers being the source of information, as was also found in another study [16]. To potentially mitigate any issues with media sources, experts in infectious diseases, health communication, and social marketing should be an integral part of antimicrobial awareness campaign planning teams [21]. This is especially important considering the potential reach of these media sources and their potential for the spread of misinformation regarding health matters [22]. Educational campaigns were also found to negatively influence knowledge scores in the multivariate regression (β = −1.4, 95% CI: −2.6, −0.17, and *p* = 0.025). The findings about media sources and education campaigns may be explained by the findings from a study assessing sources of health information among adolescents. That study highlighted that health knowledge presentations that include engaging and participatory activities were preferred over others [18]. This may explain why media and educational campaigns were negatively associated with knowledge, as they may not be as engaging and participatory as comics, especially if they are not specifically designed to be so. This may also be a case for including interactive public health education in the activities of health facilities and professionals as good sources of objective information. Especially, doing so in an engaging and interactive manner, as was done in this study, could help change knowledge and perceptions around AMR positively. Further, the reported educational campaigns may have used older content, which might have used outdated, overly simplified, or conflicting AMR terminology. Another potential reason for this finding may also be because the participants accounting for this may have had lower overall baseline scores, which may have confounded the overall results at the end of the study.

### 3.1. Strengths and Limitations

The intervention included all adolescent students in Basic School 7 to 9 (JHS 1 to 3), which may increase the power of the study while reducing errors, which is a strength of this study. This intervention may have been limited by the fact that the classes were educated at separate times, which may have exposed other classes not trained by the project team to information from other students who had received the training earlier. This could have artificially increased the scores for the pre-test compared to if they had not been exposed to the interventions at all. Additionally, no control group was used in this study, making it difficult to attribute all the observed changes to the intervention alone. Data from many of the students who took part in the intervention were not included in the analysis, as their pre- and post-test results could not be matched by the authors. This may pose a risk of selection bias in our sample; however, since all students were included in the intervention, managed the same way, and given the same instructions, the availability of data from a majority of the students may reduce this risk. The majority of students had families with a high education, which may limit the generalizability of our findings. In future iterations of our study, we could target schools in impoverished communities or place a cap on the number of students from highly educated families to mitigate this.

### 3.2. Policy Implications

The demonstrated outcome of comics in improving AMR knowledge among adolescents supports the case for integrating visual storytelling tools into national school health curricula. Given the critical need to address AMR through early education, this intervention model can inform Ghana’s Ministry of Education and Ghana Health Service policies on AMR education. Additionally, inclusion in the national AMR action plan—particularly in school-based awareness programs—could scale the impact of such low-cost, high-engagement strategies across other Basic Schools in Ghana and similar LMIC contexts. Based on these findings, a policy brief or implementation toolkit could be developed for education stakeholders, enabling replication and scalability at regional and national levels.

### 3.3. Future Research

While this study demonstrates short-term gains in knowledge and attitudes, future research should examine the long-term retention of AMR knowledge and its influence on behavior change. Follow-up assessments at 6- and 12-month intervals would help determine the durability of the intervention’s impact. In addition, studies incorporating qualitative methods (e.g., focus groups, interviews) could explore how students interpret and apply comic-based messages in daily life.

There is also a need to explore the effectiveness of comic-based interventions across diverse LMIC contexts with varying literacy levels, cultural norms, and healthcare infrastructure. Comparative studies across rural and urban schools, as well as randomized trials testing different comic formats (e.g., animation, interactive web-based versions), could optimize delivery approaches for broader implementation. Finally, evaluating the cost-effectiveness and feasibility of scaling such interventions nationally will provide important data to inform the integration into formal AMR education programs.

## 4. Methods

### 4.1. Study Setting

The University Basic Schools of KNUST provide basic education to children and adolescents of university staff as well as those from neighboring communities in the Oforikrom Municipality and beyond. Basic Schools provide educational services from creche to junior high school (JHS). The JHS students were the target of an educational campaign organized by the AMS committee of the University Hospital, KNUST. Ghana runs a three-year JHS system following six years of elementary education in preparation for a further three years of senior high school or technical and vocational training [23,24].

The University Hospital is a 135-bed district-level hospital located on the campus of the KNUST, Kumasi, within the Oforikrom municipality, with a catchment population of over 300,000. It provides general and specialist services to students of the university, staff, and their dependents, as well as private patients within the municipality and greater Kumasi [25].

The hospital has an AMS committee comprising multi-disciplinary healthcare professionals made up of three pharmacists, a nurse (who also served as the hospital’s infection prevention and control (IPC) focal person), three medical doctors (one is an infectious disease specialist), a hospital administrator, and two laboratory scientists responsible for undertaking activities in AMS.

### 4.2. Study Design

This was a comparative before–after study assessing participant knowledge and perceptions regarding AMR at a junior high school in Kumasi, Ghana. As part of the AMS committee’s 2023 World Antimicrobial Resistance Awareness Week celebrations, an awareness exercise was organized for JHS 1, 2, and 3 students at the KNUST Basic Schools. A storyline was developed by A.B.O. and illustrated into comics by M.M.B.B. in the English language. Five students, each from JHS 1, 2, and 3, were conveniently selected based on their willingness to be interviewed to review the comics developed for coherence and understanding. Each student was asked to provide a verbal summary of the comics they had reviewed and to comment on how well they understood the content. All students were able to understand the concept of AMR as well as identify inappropriate antibiotic use such as not finishing prescribed antibiotics as a driver of AMR.

The comics were then presented as a slideshow and subsequently printed hard copies to all JHS 1, 2, and 3 students present in school on the 23rd, and 30th of November and 7th of December, respectively, by a member of the AMS committee. Questionnaires were distributed among the students before and after exposure to the comics to assess the impact of the program on AMR awareness, attitude, and potential practices. Based on the content of the comics, questionnaires from previous studies were adapted to assess the knowledge and perceptions of the students regarding AMR. Printed copies of the comics were provided to all students after the last educational session had been done. A transcript of the comics are available in the Supplementary Materials.

### 4.3. Data Collection Instrument

#### 4.3.1. Questionnaire Development

Data were collected using a structured, self-administered questionnaire adapted from established Knowledge–Attitude–Practice (KAP) tools on antibiotic use and antimicrobial resistance. Questionnaire items were drawn from the World Health Organization’s multi-country public awareness survey on antibiotic resistance and from the instrument developed by Huang et al. (2013) [26,27]. These sources were chosen because they have been widely used to assess public understanding of antibiotics and antimicrobial resistance across different populations. The questionnaire was carefully reviewed and modified to ensure that the language and examples were appropriate for junior high school students, while preserving the original meaning of each item.

#### 4.3.2. Measures

The knowledge assessment consisted of 10 questions, categorized into two areas: knowledge about antibiotic resistance (four questions) and knowledge about antibiotic use (six questions). Each question related to antibiotics was awarded one mark if answered correctly and zero if incorrect. The maximum possible score for the assessment was 10, and the minimum was 0. The overall scores for each participant were computed and transformed into percentages. To categorize the knowledge, we used Bloom’s cut-off point, with 80–100% being good knowledge level, 60–79% being moderate knowledge level, and <60% and below for poor knowledge level [28].

#### 4.3.3. Data Analysis

Descriptive statistics were used to summarize the study variables; categorical variables were presented in frequency and percentages, and continuous variables were expressed using the median and interquartile ranges. To evaluate the significant difference in overall knowledge levels between the pre-test and post-test, a Stuart–Maxwell test (2 × 3) was applied, while THE McNemar test was used for individual knowledge items with dichotomous responses (2 × 2). A simple linear regression model was also used, using the difference between scores (post-test minus pre-test) as the dependent variable and the independent variables to estimate crude beta coefficients. From this approach, we were able to quantify the impact of each of the independent variables on the observed change in knowledge. After adjusting for confounding, variables with a *p*-value < 0.10 in the unadjusted analysis were included as covariates in a multivariable linear regression to obtain the adjusted beta coefficient.

Despite the study design inherently violating the assumption of independence in linear regression due to repeated measures on the same individuals, the difference in scores was used as the dependent variable to address this issue [29]. By analyzing the difference directly, we effectively reduced the within-subject correlation, focusing on the net change attributable to the independent variables. This approach assumes that individual-specific effects are implicitly controlled, since each participant serves as their own baseline, mitigating some of the dependency concerns. Significance level was set at 5%. All analyses were carried out using R Programming Language version 4.4.0 (R Core Team. 2024. “R: A Language and Environment for Statistical Computing.” https://www.R-project.org/).

## 5. Conclusions

Comic-based education was able to increase AMR knowledge scores and positive perceptions among adolescents. The students showed marked gains in understanding the message on appropriate antibiotic use and the consequences of their misuse. These findings suggest that comics are a valuable tool for enhancing AMR awareness in school settings. Integrating engaging visual storytelling into health education can promote better awareness and responsible behaviors, and this approach has the potential for broader application in similar educational contexts to help combat AMR.

## Figures and Tables

**Table 1:** Sociodemographic characteristics of participants included in the educational intervention using comics at the University Basic School, 2023.

Characteristic	N = 611 ^1^
Class	
JHS 1	228 (37.32%)
JHS 2	212 (34.70%)
JHS 3	171 (27.99%)
Age, Years	
Median (Q1, Q3)	13.00 (12.00, 13.00)
Gender	
Female	340 (55.65%)
Male	271 (44.35%)
Living Situation	
Extended Family	31 (5.23%)
Immediate Family	1 (0.17%)
Legal Guardian	4 (0.67%)
Parents	557 (93.93%)
Missing	18
Father’s Age, Years	
Median (Q1, Q3)	46 (40, 50)
Missing	9
Father’s Educational Level	
Basic	15 (2.66%)
Secondary	39 (6.91%)
Technical	82 (14.54%)
Tertiary	428 (75.89%)
Missing	47
Mother’s Age	
Median (Q1, Q3)	41 (35, 45)
Missing	10
Mother’s Educational Level	
Basic	34 (5.95%)
Secondary	74 (12.96%)
Technical	95 (16.64%)
Tertiary	368 (64.45%)
Missing	40
Guardian’s Educational Level	
Basic	1 (2.44%)
Secondary	6 (14.63%)
Technical	9 (21.95%)
Tertiary	25 (60.98%)
Missing	570

^1^ N (%), missing: this refers to data that was not provided by the respondents due to being unsure or not knowing the answers.

**Table 2:** Sources of knowledge of participants included in the educational intervention using comics at the University Basic School, 2023.

Characteristic	N = 611 ^1^
Awareness of Antibiotic Resistance	558 (94.58%)
Source of Information	
Health Professional	339 (60.75%)
Educational Campaign	166 (29.75%)
Media—TV, Radio, Social Media, etc.	66 (11.83%)
Textbook—School Curriculum	32 (5.73%)
Family	11 (1.97%)
Friend	13 (2.33%)
Knows Meaning of Antibiotic Resistance	557 (92.99%)
First-degree Family Member with Healthcare Occupation	381 (65.58%)

^1^ N (%).

**Table 3:** Participant perceptions included in the educational intervention using comics at the University Basic School, 2023.

Characteristic	Pre-Test N = 611 ^1^	Post-Test N = 611 ^1^	*p*-Value ^2^
Stopping Antibiotics When Better			<0.001
NO	223 (36.5%)	111 (18.2%)	
YES	388 (63.5%)	500 (81.8%)	
Missed Doses and Resistance			0.12
NO	267 (43.7%)	241 (39.4%)	
YES	344 (56.3%)	370 (60.6%)	
Antibiotics Useful for Viral Infections?			0.013
NO	314 (51.4%)	269 (44.0%)	
YES	297 (48.6%)	342 (56.0%)	
Unconcerned About Antibiotic Resistance			0.050
NO	177 (29.0%)	149 (24.4%)	
YES	434 (71.0%)	462 (75.6%)	
Antibiotic Resistance a Problem Beyond Ghana?			0.3
NO	165 (27.0%)	150 (24.5%)	
YES	446 (73.0%)	461 (75.5%)	
Antibiotic Resistance Has Limited Impact?			0.8
NO	219 (35.8%)	224 (36.7%)	
YES	392 (64.2%)	387 (63.3%)	
I Have a Personal Role in Reducing Antibiotic Resistance			<0.001
NO	199 (32.6%)	91 (14.9%)	
YES	412 (67.4%)	520 (85.1%)	

^1^ N (%); ^2^ McNemar’s Chi-squared test with continuity correction.

**Table 4:** Knowledge regarding antibiotic resistance among participants included in the educational intervention using comics at the University Basic School, 2023.

Characteristic	Pre-Test N = 611 ^1^	Post-Test N = 611 ^1^	*p*-Value ^2^
Antibiotic Resistance: Loss of Effectiveness			<0.001
Correct	362 (59.2%)	479 (78.4%)	
Wrong	249 (40.8%)	132 (21.6%)	
Antibiotic Resistance Risk: Leftover Medication			<0.001
Correct	291 (47.6%)	470 (76.9%)	
Wrong	320 (52.4%)	141 (23.1%)	
Antibiotic Treatment Failure: Incomplete Course			<0.001
Correct	433 (70.9%)	553 (90.5%)	
Wrong	178 (29.1%)	58 (9.5%)	
Antibiotic Resistance Risk: Self-Medication			<0.001
Correct	352 (57.6%)	511 (83.6%)	
Wrong	259 (42.4%)	100 (16.4%)	

^1^ N (%); ^2^ McNemar’s Chi-squared test with continuity correction.

**Table 5:** Knowledge regarding antibiotic use among participants included in the educational intervention using comics at the University Basic School, 2023.

Characteristic	Pre-Test N = 611 ^1^	Post-Test N = 611 ^1^	*p*-Value ^2^
Familiar with Antibiotics			<0.001
Correct	267 (43.7%)	579 (94.8%)	
Wrong	344 (56.3%)	32 (5.2%)	
Antibiotics Do Not Treat Viruses			<0.001
Correct	210 (34.4%)	343 (56.1%)	
Wrong	401 (65.6%)	268 (43.9%)	
Early Antibiotics Prevent Infection?			0.037
Correct	171 (28.0%)	201 (32.9%)	
Wrong	440 (72.0%)	410 (67.1%)	
Antibiotics for Cough and Wheezing?			<0.001
Correct	243 (39.8%)	364 (59.6%)	
Wrong	368 (60.2%)	247 (40.4%)	
Low-Dose Antibiotics Beneficial?			0.007
Correct	234 (38.3%)	274 (44.8%)	
Wrong	377 (61.7%)	337 (55.2%)	
Antibiotic-Resistant Bacteria Spread			<0.001
Correct	283 (46.3%)	424 (69.4%)	
Wrong	328 (53.7%)	187 (30.6%)	

^1^ N (%); ^2^ McNemar’s Chi-squared test with continuity correction.

**Table 6:** Overall knowledge level of antibiotic resistance and use among participants included in the educational intervention using comics at the University Basic School, 2023.

Knowledge Level	Pre-Test N = 611 ^1^	Post-Test N = 611 ^1^	*p*-Value ^2^
Good	29 (4.75%)	181 (29.62%)	<0.001
Moderate	171 (27.99%)	250 (40.92%)	
Poor	411 (67.27%)	180 (29.46%)	

^1^ N (%); ^2^ Stuart–Maxwell test.

**Table 7:** Simple linear regression in determinants of knowledge on antimicrobial and antimicrobial resistance among participants at the University Basic School, 2023.

Characteristic	N	Beta	95% CI ^1^	*p*-Value
Class	611			
JHS 1		Reference	Reference	
JHS 2		0.66	0.24, 1.1	0.002
JHS 3		0.01	−0.42, 0.45	0.948
Age, Years	611	−0.04	−0.20, 0.13	0.663
Gender	611			
Female		Reference	Reference	
Male		−0.67	−1.0, −0.32	<0.001
Father’s Age, Years	591	−0.01	−0.02, 0.00	0.108
Father’s Educational Level	551			
Basic		Reference	Reference	
Secondary		0.68	−0.56, 1.9	0.283
Technical		0.34	−0.77, 1.5	0.545
Tertiary		0.67	−0.37, 1.7	0.208
Mother’s Age	596	−0.01	−0.02, 0.00	0.166
Mother’s Educational Level	548			
Basic		Reference	Reference	
Secondary		−0.10	−0.98, 0.78	0.830
Technical		−0.13	−1.0, 0.73	0.764
Tertiary		0.03	−0.75, 0.81	0.939
Staying With Biological Parents	611			
Staying With Biological Parents		Reference	Reference	
No		0.60	−0.79, 2.0	0.395
Yes		0.37	−0.82, 1.6	0.542
Living Situation	597			
Extended Family		Reference	Reference	
Immediate Family		0.07	−2.6, 2.7	0.960
Legal Guardian		1.1	−1.6, 3.7	0.432
Parents		−0.13	−0.97, 0.70	0.754
Source of Information (Health Professional)	244			
No		Reference	Reference	
Yes		−0.01	−0.54, 0.51	0.958
Source of Information (Educational Campaign)	244			
No		Reference	Reference	
Yes		−1.2	−2.4, 0.03	0.056
Source of Information (Media—TV, Radio, Social Media, etc.)	244			
No		Reference	Reference	
Yes		−0.57	−1.1, −0.05	0.033
Source of Information (Textbook—School Curriculum)	244			
No		Reference	Reference	
Yes		0.03	−0.65, 0.71	0.931
Source of Information (Family)	244			
No		Reference	Reference	
Yes		0.02	−0.96, 1.0	0.971
Source of Information (Friend)	244			
No		Reference	Reference	
Yes		0.90	−0.13, 1.9	0.086
First-degree Family Member With Healthcare Occupation	579			
No		Reference	Reference	
Yes		0.00	−0.37, 0.36	0.994

^1^ CI = Confidence Interval.

**Table 8:** Multivariable linear regression analysis on determinants of knowledge on antimicrobial and antimicrobial resistance among participants at the University Basic School, 2023.

Characteristic	Beta	95% CI ^1^	*p*-Value
Class			
JHS 1	Reference	Reference	
JHS 2	0.57	−0.04, 1.2	0.065
JHS 3	0.25	−0.38, 0.87	0.437
Gender			
Female	Reference	Reference	
Male	−0.51	−1.0, −0.01	0.045
Source of Information (Media—TV, Radio, Social Media, etc.)			
No	Reference	Reference	
Yes	−0.56	−1.1, −0.04	0.035
Source of Information (Educational Campaign)			
No	Reference	Reference	
Yes	−1.4	−2.6, −0.17	0.025
Source of Information (Friend)			
No	Reference	Reference	
Yes	0.85	−0.18, 1.9	0.107

^1^ CI = Confidence Interval.

## Data Availability

The data presented in this study are available on request from the corresponding author. (data are not made public due to the privacy of the study participants who are adolescents).

## Supplementary Materials

The following supporting information can be downloaded at: https://www.mdpi.com/article/10.3390/antibiotics15070646/s1. Infographics for Basic School (Storyline).

## Abbreviations

AMS	Antimicrobial Stewardship
AMR	Antimicrobial Resistance
KNUST	Kwame Nkrumah University of Science and Technology
JHS	Junior High School
IPC	Infection Prevention and Control
KAP	Knowledge Attitude Practice
CwPAMS	Commonwealth Partnerships for Antimicrobial Stewardship
CPA	Commonwealth Pharmacists Association
GHP	Global Health Partnerships
ODA	Official Development Assistance

## References

[1] Laxminarayan R., Duse A., Wattal C., Zaidi A.K., Wertheim H.F., Sumpradit N., Vlieghe E., Hara G.L., Gould I.M., Goossens H. (2013). Antibiotic resistance-the need for global solutions. Lancet Infect. Dis..

[2] World Health Organization (2019). WHO|Antimicrobial Resistance. WHO. https://www.who.int/antimicrobial-resistance/en/.

[3] Prestinaci F., Pezzotti P., Pantosti A. (2015). Antimicrobial resistance: A global multifaceted phenomenon. Pathog. Glob. Health.

[4] Hayes C.V., Eley C.V., Wood F., Demirjian A., McNulty C.A.M. (2021). Knowledge and attitudes of adolescents towards the human microbiome and antibiotic resistance: A qualitative study. JAC-Antimicrob. Resist..

[5] McNulty C.A.M., Boyle P., Nichols T., Clappison P., Davey P. (2007). Don’t wear me out—The public’s knowledge of and attitudes to antibiotic use. J. Antimicrob. Chemother..

[6] Hawking M.K., Lecky D.M., Touboul Lundgren P., Aldigs E., Abdulmajed H., Ioannidou E., Paraskeva-Hadjichambi D., Khouri P., Gal M., Hadjichambis A.C. (2017). Attitudes and behaviours of adolescents towards antibiotics and self-care for respiratory tract infections: A qualitative study. BMJ Open.

[7] Freedman D.S., Khan L.K., Dietz W.H., Srinivasan S.R., Berenson G.S. (2001). Relationship of childhood obesity to coronary heart disease risk factors in adulthood: The Bogalusa Heart Study. Pediatrics.

[8] Lazzeri G., Azzolini E., Pammolli A., Simi R., Meoni V., Giacchi M.V. (2014). Factors associated with unhealthy behaviours and health outcomes: A cross-sectional study among Tuscan adolescents (Italy). Int. J. Equity Health.

[9] O’Loughlin J.L., Tarasuk J. (2003). Smoking, Physical Activity, and Diet in North American Youth. Can. J. Public Health.

[10] Tabrizi J.S., Doshmangir L., Khoshmaram N., Shakibazadeh E., Abdolahi H.M., Khabiri R. (2024). Key factors affecting health promoting behaviors among adolescents: A scoping review. BMC Health Serv. Res..

[11] Ventola C.L. (2015). The Antibiotic Resistance Crisis: Part 1: Causes and Threats. Pharm. Ther..

[12] Nascimento G.B.S., de Maio Nascimento M., de Araújo L.M.G., Gouveia É.R., Ihle A. (2023). Comics as a Physical Education Tool for Health Promotion in Brazilian Primary Education, Based on Paulo Freire’s Principles of Empowerment. Children.

[13] Soares A., Santos A.B.M.V.D., Vieira T.S.D., Xavier B.L.D.Q., Lucas R.J.D.L., Oliveira Â.G.R.D.C. (2024). Use of comics in the promotion of school children’s health: A scoping review. Front. Commun..

[14] King A.J. (2017). Using Comics to Communicate About Health: An Introduction to the Symposium on Visual Narratives and Graphic Medicine. Health Commun..

[15] MacDougall C., Polk R.E. (2005). Antimicrobial stewardship programs in health care systems. Clin. Microbiol. Rev..

[16] Dutta-Bergman M. (2003). Trusted online sources of health information: Differences in demographics, health beliefs, and health-information orientation. J. Med. Internet Res..

[17] Chan A.H.Y., Horne R., Lycett H., Raebel E., Guitart J., Wildman E., Ang K. (2021). Changing Patient and Public Beliefs About Antimicrobials and Antimicrobial Resistance (AMR) Using a Brief Digital Intervention. Front. Pharmacol..

[18] Racey M., Machmueller D., Field D., Kulak V., Newton G.S. (2018). Perceptions and use of sources of health knowledge by young adolescents. Int. J. Adolesc. Med. Health.

[19] Van Houtte M. (2004). Why boys achieve less at school than girls: The difference between boys’ and girls’ academic culture. Educ. Stud..

[20] Warrington M., Younger M., Williams J. (2000). Student attitudes, image and the gender gap. Br. Educ. Res. J..

[21] Huttner B., Saam M., Moja L., Mah K., Sprenger M., Harbarth S., Magrini N. (2019). How to improve antibiotic awareness campaigns: Findings of a WHO global survey. BMJ Glob. Health.

[22] Suarez-Lledo V., Alvarez-Galvez J. (2021). Prevalence of Health Misinformation on Social Media: Systematic Review. J. Med. Internet Res..

[23] Adu-Agyem J., Osei-Poku P. (2012). Quality education in Ghana: The way forward. Int. J. Innov. Res. Dev..

[24] Anon Educational System of Ghana—U.S. Embassy in Ghana. https://gh.usembassy.gov/education-culture/educationusa-center/educational-system-ghana/.

[25] Anon About Us|University Health Services. https://uhs.knust.edu.gh/about-us.

[26] WHO (2015). Multi-Country Public Awareness Survey.

[27] Huang Y., Gu J., Zhang M., Ren Z., Yang W., Chen Y., Fu Y., Chen X., Cals J.W., Zhang F. (2013). Knowledge, attitude and practice of antibiotics: A questionnaire study among 2500 Chinese students. BMC Med. Educ..

[28] Seid M.A., Hussen M.S. (2018). Knowledge and attitude towards antimicrobial resistance among final year undergraduate paramedical students at University of Gondar, Ethiopia. BMC Infect. Dis..

[29] Allison P.D. (1990). Change Scores as Dependent Variables in Regression Analysis. Sociol. Methodol..

