# Supplementary material for: Comic-Based Educational Intervention Enhances Antimicrobial Resistance Knowledge and Perceptions Among Adolescents in Ghana
Source: Antibiotics (Basel). 2026 Jun 29;15(7):646. doi: 10.3390/antibiotics15070646 (PMC13403375; doi:10.3390/antibiotics15070646)
Supplement: Supplementary file 1 [file antibiotics-15-00646-s001.zip › antibiotics-4346647-supplementary.pdf]

INFOGRAPHICS FOR BASIC SCHOOL (STORYLINE)

Slide 1: The Microbe Menace

- Illustration of a friendly city with kids and their pets.
- An evil microbe villain arrives, threatening the city.

Slide 2: Heroes to the Rescue

- Introduce the heroes, Dr. Antibiotic and Captain Pharmacist.
- Dr. Antibiotic has a magic shield representing antibiotics.
- Captain Pharmacist has a bag of medicine.

Slide 3: The Microbe's Weakness

- Show the microbe villain's weaknesses (mutation, infection).
- Dr. Antibiotic's shield can defeat the microbe for now.

Slide 4: Misuse of Antibiotics

- Illustrate kids taking antibiotics for the wrong reasons (colds, viruses).
- Explain that antibiotics don't work against viruses.

Slide 5: The Rise of Superbugs

- Show the microbe villain evolving into a superbug.
- Explain that overuse of antibiotics causes superbugs.

Slide 6: Captain Pharmacist's Mission

- Captain Pharmacist teaches kids about using medicine wisely.
- Explain the importance of following prescription instructions.

Slide 7: Dr. Antibiotic's Dilemma

- Dr. Antibiotic's shield is weakening against the superbug.
- Explain how superbugs are resistant to antibiotics. (Superbug wins)

Slide 8: The Power of Antibiotic Stewardship

- Show kids and parents using antibiotics responsibly.
- Illustrate a happy, healthy city as a result.

Slide 9: Our Pledge

- Encourage kids to take the pledge to use antibiotics wisely.

Slide 10: Victory Celebration

- Show the heroes celebrating a healthy city.
- Kids and their pets join the celebration.

Slide 11: Superbug Defeated!

- The microbe villain is now a tiny, harmless microbe.
- Explain that responsible antibiotic use saved the day.

Slide 12: The End

- Thank kids for being antibiotic heroes.

PLEDGE

I pledge to be a guardian of our health and a protector of our future.

I promise to learn and understand the importance of antibiotics and how they can help us when we are sick.

I vow to use antibiotics only when a healthcare professional prescribes them for me.

I commit to completing the entire course of antibiotics as prescribed, even if I start feeling better.

I will share this knowledge with my friends and family, spreading the word about responsible antibiotic use.

Together, we can make a difference in the fight against Antimicrobial Resistance. We are the Young Guardians Against Superbugs!
